# A comparative laboratory trial evaluating the immediate efficacy of fluralaner, afoxolaner, sarolaner and imidacloprid + permethrin against adult *Rhipicephalus sanguineus* (*sensu lato*) ticks attached to dogs

**DOI:** 10.1186/s13071-016-1900-z

**Published:** 2016-12-03

**Authors:** Federica Burgio, Leon Meyer, Rob Armstrong

**Affiliations:** 1 MSD Animal Health, Josefa Valcarcel 38, 28080 Madrid, Spain; 2 Clinvet International, Uitzich Road, Bainsvlei 9338, Bloemfontein, South Africa; 3 MSD Animal Health, 2 Giralda Farms, Madison, NJ 07940 USA

**Keywords:** Acaricide, Imidacloprid, Permethrin, Fluralaner, Afoxolaner, Sarolaner, Dog, Tick, *Rhipicephalus sanguineus*

## Abstract

**Background:**

Acaricides are used to treat and prevent tick infestations, and a common clinical scenario is to administer an acaricide on observing an attached tick. Consequently, immediate acaricidal efficacy (onset of activity and speed of kill) results are clinically valuable. This study evaluated the immediate efficacy of four commercially available acaricides against adult *Rhipicephalus sanguineus* (*sensu lato*).

**Methods:**

Forty dogs were blocked on hair length and tick carrying capacity, then randomly assigned to receive one of four treatments (fluralaner, sarolaner, imidacloprid + permethrin, or afoxolaner) or left untreated as controls. All dogs were challenged with 50 adult *R. sanguineus* (*s.l*.) ticks 48 h prior to treatment. After treatment, *in situ* tick thumb counts were conducted at 2, 4, 8, 12 and 24 h; thereafter ticks were removed and counted at 48 h.

**Results:**

Imidacloprid + permethrin had the earliest onset of activity at 2 h (36.9% efficacy) followed at 4 h by fluralaner (60.2% efficacy) and sarolaner (48.2% efficacy), and lastly afoxolaner at 8 h (90.8% efficacy). Three oral treatments had an 8 h speed of kill (>90% efficacy) threshold; with corresponding efficacies as: fluralaner (99.6%), sarolaner (94.7%) and afoxolaner (90.8%). Fluralaner and sarolaner achieved 100% efficacy at 12, 24 and 48 h; afoxolaner achieved 100% efficacy at 48 h. Imidacloprid + permethrin achieved 80.1% efficacy at 48 h, therefore, failing to attain the speed of kill 90% efficacy threshold.

**Conclusion:**

The systemically distributed isoxazolines performed much better than cutaneously distributed imidacloprid + permethrin and are optimal treatment choices against attached ticks based on the combination of earlier onset of activity and speed of kill. Fluralaner had a 4 h onset of activity, an 8 h speed of kill and achieved 100% efficacy at 12 h.

## Background

Even with the availability of safe and potent acaricides, the possibility of attached ticks on dogs is always a concern, perhaps because of weak dog owner adherence to product administration directions [1–3]. Attached ticks present a direct threat to dogs from site irritation, blood loss and anaemia from feeding, as well as presenting potentially fatal indirect threats from pathogen transmission. Therefore, an appropriate response following observation of an attached tick on a dog is to carefully remove the tick and administer an acaricidal agent against other attached, but unobserved, ticks. There is some urgency in tick removal, because there is a ‘window of opportunity’ following tick attachment before pathogen transmission occurs and reducing the attachment time could also reduce the risk of pathogen transmission [4].

This study was designed to measure the immediate efficacy, defined as efficacy against ticks potentially attached at the time of treatment [5], of four different acaricides. The immediate acaricidal efficacy profile can include different activity measurements including: onset of activity, speed of kill and onset of effect. ‘Onset of activity’ is used to refer to the earliest time that significant parasite killing activity can be shown after treatment administration [6] and therefore is the first treatment threshold reached. Demonstration of a significant difference between treated and untreated animals is the critical result needed to establish the onset of activity although significance can be achieved with comparatively low observed efficacy if a sufficiently large sample size is used. An early ‘onset of activity’ has value in providing dog owners with rapid evidence of activity against attached ticks. ‘Speed of kill’ refers to the time required to achieve a defined efficacy threshold (usually > 90% for ticks) and can be measured at any elapsed time point after treatment administration, therefore, the elapsed time after treatment should be specified with the result [7]. Speed of kill has clinical relevance because it specifies the time to reach the accepted efficacy threshold. A positive correlation between immediate efficacy and a reduced tick borne pathogen transmission risk is logical, but is not quantified, and even 100% immediate efficacy may not completely preclude pathogen transmission under some circumstances. A third measurement - often included in approved product labels in Europe - is the ‘onset of effect’, which refers to the longest time required to reach the speed of kill efficacy level (90% efficacy for ticks) during the recommended treatment interval [8]. ‘Onset of effect’ is not usually a characteristic of immediate treatment efficacy because speed of kill is likely to be slowest toward the end of the treatment interval.

Both systemically and cutaneously distributed acaricides are available [8]. Permethrin, a commonly used synthetic pyrethroid, is applied topically and distributed cutaneously, and was applied in this study as the combination product imidacloprid + permethrin. Permethrin is the only component in this combination with acaricidal or repellent activity [9]. Fluralaner, afoxolaner and sarolaner are recently introduced isoxazoline acaricides, and are administered orally and distributed systemically. Systemically distributed acaricides can kill attached ticks at any location on the body when the tick feeds [8, 10]; therefore, systemically distributed isoxazolines may be expected to have better immediate efficacy, because they are quickly and widely distributed in the blood circulation. Cutaneously distributed acaricides depend on diffusion to spread over the body surface on the skin, typically in the lipid layer [8]. Comparative immediate efficacy results have not previously been reported for these four commercially available acaricides.

The tick selected for this study was the brown dog tick *Rhipicephalus sanguineus* (Latreille, 1806) (*sensu lato)* [11] a three-host tick that feeds primarily on dogs and will occasionally attach to other animals, including humans [12, 13]. *Rhipicephalus sanguineus* (*s.l*.) is probably the most widely distributed tick species in the world and is linked to tick-borne pathogen transmission to both dogs and humans, including *Babesia spp.*, *Ehrlichia spp.*, and *Rickettsia* spp. [5, 12–14]. *Rhipicephalus sanguineus* ticks are endophilic, i.e. they are often found indoors, while most other ixodid ticks tend to complete their life-cycles outdoors [12, 13] and are negatively geotactic and can be found climbing walls, floors and furniture in infested residences [12, 13]. *Rhipicephalus sanguineus* is active all year in warmer climatic zones, and is an important cause of infestation in the Mediterranean area [12] and apparently has been for millennia as it was identified on a mummified Egyptian dog [15].

The objective of this study was to evaluate the immediate efficacy of four commercially available acaricides against adult *R. sanguineus* (*s.l*.) to provide valuable information to veterinarians and dog owners faced with the situation of treating a dog presented with attached ticks.

## Methods

This was a single centre, parallel group, randomised block design efficacy study, with the investigator blinded to treatment status. The study was conducted under the approval of the Institutional Animal Care and Use Committee (IACUC) at Clinvet International and IACUC members had the authority to inspect the study site and the animals at will. Initially, 48 dogs that had not been treated with a systemic or topical insecticide within the previous 12 weeks were selected, confirmed as healthy based on physical examination and then acclimatized on site. Forty of the 48 selected dogs were included in the study based on their tick carrying capacity in a preliminary 48 h challenge conducted 14 days before treatment administration. These 40 dogs were ranked within average hair length groups (short < 30 mm and medium 30–50 mm), and then ranked in descending order of pre-treatment live attached *R. sanguineus* (*s.l*.) counts to create eight blocks of five dogs each. Within blocks, dogs were randomly allocated to five treatment groups. To achieve blinding, dogs were randomly assigned to coded treatment groups by a non-blinded person. Hair length blocking was used to reduce the chance of introducing an accidental bias against the cutaneously distributed treatment, which might be more challenging to apply and therefore have a slightly slower distribution in a long haired dog. Dogs were individually housed during the study and received regular nutrition and water.

For tick challenges, dogs were sedated for approximately 1 h and placed in an infestation chamber. A balanced sex ratio (50% female: 50% male) of 50 unfed adult ticks that were at least one week old from a laboratory-bred strain of *R. sanguineus* (*s.l*.) (USA strain) were used in infestation challenges. Tick challenges were applied below an imaginary line drawn on the lateral side of the animal’s body from the shoulder in a straight line to the greater trochanter of the hip until the buttocks with the infestation site as close as possible to the hind leg; however, the ticks could potentially attach to any location on the dog. This approach was used because attached ticks might be found at many sites on the dog and therefore it would reduce an efficacy bias that could occur if ticks were exclusively applied to the back of the dog close to the administration site of a cutaneously distributed treatment. *Rhipicephalus sanguineus* can attach anywhere but apparently prefer the ears, interdigital spaces, back, inguinal region and axillae [13].

The four treatment groups received sarolaner (Simparica, Zoetis, Florham Park, NJ, USA), afoxolaner (NexGard, Merial, Lyon, France), fluralaner (Bravecto, MSD Animal Health, Madison, NJ, USA) or imidacloprid + permethrin (K9 Advantix, Bayer Animal Health, Leverkusen, Germany). All dogs were weighed four days before treatment and then treatments were administered to dogs in the appropriate groups according to each product’s label recommendations. Orally administered products were placed on the back of the tongue and then the mouth was checked to ensure the treatment had been swallowed. The spot-on treatment was applied at one or four spots along the back according to the dog’s weight, as specified in the package insert recommendations and dogs were briefly restrained following treatment to prevent any drip off. Dogs were observed hourly for at least 4 h after treatment, to monitor for possible adverse effects and then daily for the balance of the study period.

*In situ* (thumb counts) tick counts were conducted at 2, 4, 8, 12 and 24 h after treatment, and a removal tick count was conducted at 48 h after treatment. Treatments and infestations were co-ordinated at staggered time points to allow sufficient time to complete all tick counts. Treatment administration and tick infestation times for each dog were recorded to ensure that tick counting and then removal occurred at specified target times. Ticks were detected in the *in situ* counts by direct observation using hair coat parting and palpation for 15 min. The final removal count at 48 h was performed by using up to 50 combs with a fine tooth comb. Following the 48 h removal counts, each dog received a further combing to ensure that all ticks had been counted and removed. Removed ticks were classified as either ‘live’ or ‘dead’ and either ‘free’ or ‘attached’ creating four possible classifications. These classifications were used to calculate the efficacy in two ways, because different efficacy calculation methods have been proposed for systemic and cutaneous treatments [5]. Briefly, efficacy calculations that include live free ticks may not be appropriate for evaluating systemic treatments that require the tick to attach in order to be exposed to the active ingredient.

Efficacy against ‘live attached’ ticks = 100 × (Mc – Mt)/Mc, where:

Mc = Mean number of live attached ticks on animals in the untreated control group at a specific time point;

Mt = Mean number of live attached ticks on animals in each treated group at the same specific time point.

Efficacy against ‘live attached and free’ ticks = 100 × (Mc – Mt)/Mc, where:

Mc = Mean number of live free and attached ticks on animals in the untreated control group at a specific time point;

Mt = Mean number of live free and attached ticks on animals in each acaricide treated group at the same specific time point.

The experimental unit for the statistical analysis is the individual dog. Groups were compared using an ANOVA (Proc GLM procedure in SAS Version 9.3 TS Level 1 M2) with a treatment effect on both untransformed and logarithmic transformed tick (count + 1) data. The level of significance of the formal tests was set at 5% and all tests were two sided. The treatment was considered to have reached the 90% efficacy threshold only if the untreated control group infestation was also considered adequate with tick retention greater than 20%, or 10 of the 50 challenge ticks [5]. All treatments were compared with each other for each of the two different treatment calculation measures.

## Results

**Fig. 1:**
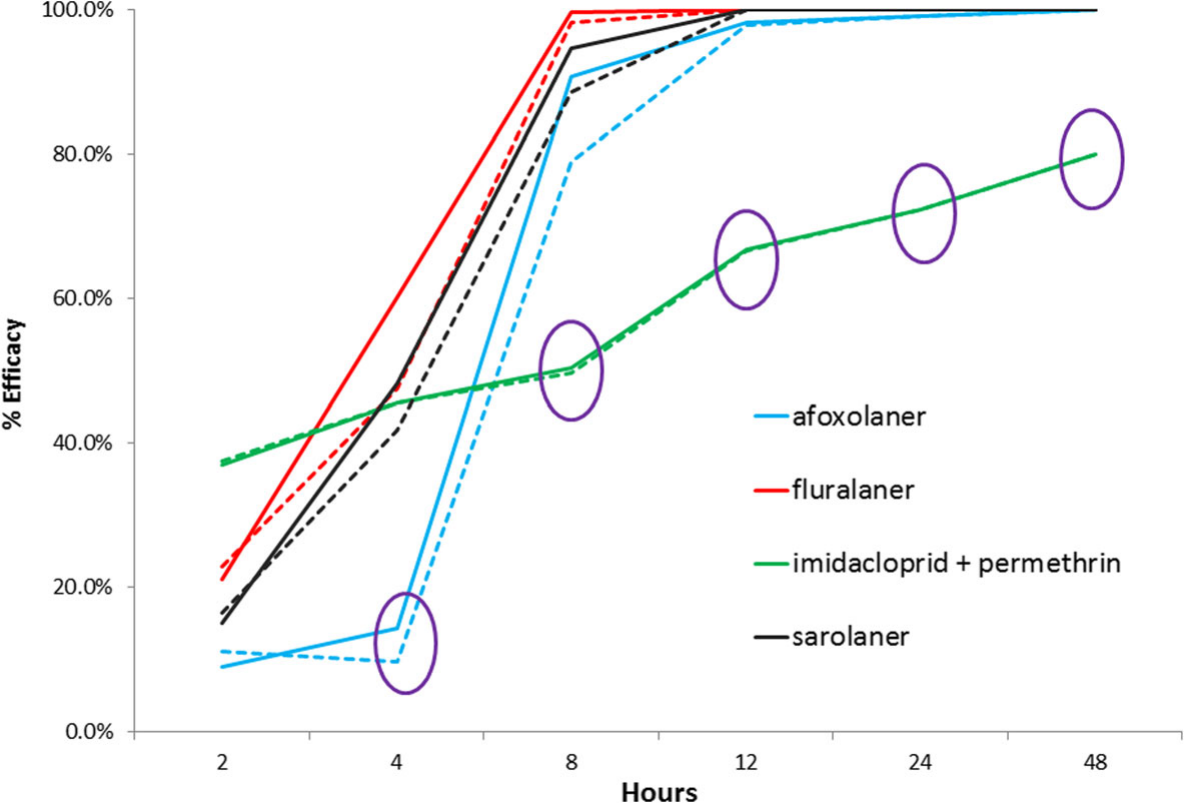
Efficacy, calculated by two methods, for four acaricidal treatments against attached adult *R. sanguineus* (*s.l*.) ticks on dogs over the 48 h following initial treatment administration. Circled results are significantly lower than all other results for the specific time point. The solid line is the efficacy calculated using ‘live attached’ ticks and the dashed line is the efficacy calculated using ‘live attached and free’ ticks. One-way ANOVA results (lowest results compared to next lowest) are consistently afoxolaner *vs* imidacloprid + permethrin: at 4 h, *P* = 0.0128 (live attached efficacy); at 8, 12, 24 and 48 h, *P* < 0.0001. For ‘live attached and free’ efficacy, one-way ANOVA results at 4 h for afoxolaner *vs* sarolaner: *P* = 0.0073

All dogs completed the study with no reported treatment related adverse effects in any group. Tick infestation adequacy was reached in the control group at every time point (Table 1). Calculated ‘Live attached’ Tick efficacy results and ‘Live attached and free’ Tick efficacy results (Fig. 1) are shown for the 48 h period with results that are significantly lower at each time point circled in the figure and one-way ANOVA *P*-values provided in the legend. ‘Onset of activity’ and ‘speed of kill’ for each treatment group at each tick count time point were determined from the efficacy results (Table 2). Total live ticks attached to treated dogs declined over the 48 h period in all treatment groups and reached 0 in all treated groups except for the imidacloprid + permethrin group (Table 3).

**Table 1:** Live attached *R. sanguineus* (*s.l*.) tick counts and infestation adequacy on untreated control group dogs (*n* = 8) throughout the 48 h treatment evaluation period that started 48 h after the initial tick challenge

Time (h) post treatment administration to the treated groups	Live attached ticks (Mean ± SD)	Live attached ticks as % of the 50 challenge ticks per dog	Control group tick infestation level adequate (>20%)?
2	26.8 ± 6.8	40–67	Yes
4	28.3 ± 4.9	47–66	Yes
8	28.5 ± 4.8	47–67	Yes
12	29.8 ± 5.3	49–70	Yes
24	29.8 ± 5.5	50–71	Yes
48	33.3 ± 6.8	53–80	Yes

*Abbreviation*: *SD* standard deviation

**Table 2:** Time to onset of activity and speed of kill using two different efficacy calculation methods for acaricidal treatments over the 48 h following administration to dogs infested with adult *R. sanguineus* (*s.l*.) 48 h prior to treatment

Treatment	Time to onset of activity (h)	Efficacy at onset time (%)	Speed of kill (time to 90% “live attached” efficacy) (h)	Efficacy (“live attached”) at “speed of kill” time point (%)	Speed of kill (time to 90% “live attached and free” efficacy) (h)	Efficacy (“live attached and free”) at “speed of kill” time point (%)	Time to 100% efficacy (the time was the same for both calculation types) (h)
Imidacloprid + Permethrin	2	36.9	Not attained	Not attained	Not attained	Not attained	Not attained
Fluralaner	4	60.2	8	99.7	8	98.2	12
Sarolaner	4	48.2	8	94.7	12	100	12
Afoxolaner	8	90.8	8	90.8	12	97.9	48

**Table 3:** The total number of live attached *R. sanguineus* (*s.l*.) ticks at each assessment time point over the 48 h following treatment administration on dogs (*n* = 8) treated with four different acaricides

Treatment/Assessment time point	2 h	4 h	8 h	12 h	24 h	48 h
Afoxolaner	195	194	21	4	2	0
Fluralaner	169	90	1	0	0	0
Imidacloprid + Permethrin	135	123	113	79	66	53
Sarolaner	182	117	12	0	0	0

Permethrin had the earliest onset of activity at 2 h with a comparatively low efficacy level that increased very slowly over the 48 h study period (Table 2, Fig. 1). Of the orally administered treatments, fluralaner and sarolaner had the earliest onset of activity at 4 h. Afoxolaner had a comparatively slower 8 h onset of activity. All of the orally administered treatments achieved the 90% speed of kill threshold by 8 h post-administration (in order of decreasing efficacy: fluralaner 99.6%; sarolaner 94.7%; afoxolaner 90.8%). The potential clinical impact of these efficacy differences is illustrated by comparing the total live tick counts on all dogs from each group at 8 h when there were: 113 attached live ticks counted on the imidacloprid + permethrin group dogs; 21 live ticks on the afoxolaner group dogs; 12 live ticks on the sarolaner group dogs; and one live tick on the fluralaner group dogs (Table 3).

All orally administered products achieved 100% efficacy - using both the ‘live attached ticks’ and ‘live attached and free ticks’ calculation techniques - during the study (fluralaner and sarolaner at 12 h and afoxolaner at 48 h) (Fig. 1). The topically distributed imidacloprid + permethrin attained a peak efficacy of only 81% during the 48 h study; therefore, not reaching the 90% speed of kill threshold (Table 2, Fig. 1). In the imidacloprid + permethrin group there were live ticks attached to dogs at every time point in the study and 53 ticks remained attached to dogs in the group at the end of the 48 h study period, while there were no ticks attached to any dog in the fluralaner and sarolaner groups after 8 h and in the afoxolaner group at 48 h (Table 3).

## Discussion

The objective of this study was to evaluate the immediate efficacy of four available acaricides that might be administered in the common clinical scenario where a veterinarian or owner treats a dog observed to have an attached tick. These results show that the orally administered, systemically distributed isoxazolines deliver significantly better immediate efficacy compared to cutaneously distributed permethrin (Table 2, Fig. 1) based on their speed of kill.

The differences in the mechanism of active ingredient distribution and the potency of the acaricidal effect for each active are likely the critical factors explaining efficacy differences observed between the groups. This study did not evaluate repellency, because tick infestation challenges were initiated 48 h before treatment administration and permethrin efficacy typically requires approximately 7 days after administration to reach peak levels [8, 16]. Systemic distribution provides a rapid conduct of active ingredient to all potential sites of tick attachment, and is critical for delivering immediate efficacy. In contrast, the immediate efficacy of cutaneously distributed treatments can be reduced by multiple factors including: the time required for distribution to body locations distant to the site of application; lack of uniform spread; and, potential for loss from the skin surface [8]. Prescribing information for permethrin + imidacloprid advises that this combination can take 48 h to kill ticks attached at the time of treatment [17]; however, this may be overly optimistic for *R. sanguineus* (*s.l*.) because in this study a total of 53 live ticks remained attached to dogs in this treatment group at the end of the 48 h.

The timing of pathogen transmission following attachment of an infected tick is variable, with a general suggestion that feeding for 24 to 48 h may be needed [4, 18]. Specific impacts of acaricides on tick feeding mechanisms and the consequent effects on tick borne pathogen transmission times were not measured in this study. Previous research has shown that each treatment administered in this study can prevent pathogen transmission under specific laboratory conditions [18–20]. If there is a direct correlation between tick feeding time and pathogen transmission, then results of this study indicate that there will be differences between the ability of the acaricides tested to prevent pathogen transmission from ticks attached at the time of treatment. A treatment that allows more ticks to remain attached for longer (as seen with the imidacloprid + permethrin group in this study) will be associated with a potentially greater risk of transmission of infectious disease pathogens. The ability of acaricides to prevent pathogen transmission under specific laboratory conditions [18–21] is not proof that they eliminate the risk of pathogen transmission under field conditions because the multiple variables occurring in a natural setting cannot be mimicked in a laboratory trial [4, 5]. Furthermore, poor dog owner compliance with parasite treatment recommendations [1–3] is another factor that reduces the ability of commercially available acaricides to control the risk of tick borne parasite transmission. Therefore, an extended persistent duration of acaricidal efficacy against *R. sanguineus* (*s.l*.) may be as important as immediate efficacy.

Two different efficacy calculations were used in this study because ‘live attached’ efficacy has been proposed as a more relevant measure for systemically distributed treatments while ‘live attached and free’ efficacy may be a more relevant measure for cutaneously distributed treatments [5]. The rationale is that systemically distributed treatments will have no effect on ticks that have not fed, while cutaneously distributed treatments could have an effect on free ticks [5]. However, there was little difference seen between the results of the two efficacy calculations in this study (Fig. 1) except for the afoxolaner group which showed a distinct dip in efficacy at 4 h post-treatment using the ‘live attached and free’ efficacy calculation. Therefore, either calculation method is a reasonable choice in assessing immediate efficacy.

## Conclusions

In conclusion, administration of a systemically distributed acaricide is a much more effective approach than a cutaneously distributed acaricide for treating a dog infested with live attached ticks. Fluralaner had a 4 h onset of activity, an 8 h speed of kill and achieved 100% efficacy at 12 h, and is an optimal choice for treating dogs with attached ticks.

## References

[1] Matos M, Alho AM, Owen SP, Nunes T, de Carvalho LM (2015). Parasite control practices and public perception of parasitic diseases: a survey of dog and cat owners. Prev Vet Med.

[2] Pereira A, Martins A, Brancal H, Vilhena H, Silva P, Pimenta P (2016). Parasitic zoonoses associated with dogs and cats: a survey of Portuguese pet owners’ awareness and deworming practices. Parasit Vectors.

[3] Leschnik M, Feiler A, Duscher GG, Joachim A (2013). Effect of owner-controlled acaricidal treatment on tick infestation and immune response to tick-borne pathogens in naturally infested dogs from Eastern Austria. Parasit Vectors.

[4] Kidd L, Breitschwerdt EB (2003). Transmission times and prevention of tick-borne diseases in dogs. Compend Contin Educ Pract Vet.

[5] Marchiondo AA, Holdsworth PA, Fourie LJ, Rugg D, Hellmann K, Snyder DE (2013). World Association for the Advancement of Veterinary Parasitology (W.A.A.V.P.) second edition: guidelines for evaluating the efficacy of parasiticides for the treatment, prevention and control of flea and tick infestations on dogs and cats. Vet Parasitol.

[6] Taenzler J, Wengenmayer C, Williams H, Fourie J, Zschiesche E, Roepke RKA (2014). Onset of activity of fluralaner (BRAVECTO™) against *Ctenocephalides felis* on dogs. Parasit Vectors.

[7] Marchiondo AA, Holdsworth PA, Green P, Blagburn BL, Jacobs DE (2007). World Association for the Advancement of Veterinary Parasitology (W.A.A.V.P.) guidelines for evaluating the efficacy of parasiticides for the treatment, prevention and control of flea and tick infestation on dogs and cats. Vet Parasitol.

[8] Pfister K, Armstrong R (2016). Systemically and cutaneously distributed ectoparasiticides: a review of the efficacy against ticks and fleas on dogs. Parasit Vectors.

[9] Beugnet F, Franc M (2012). Insecticide and acaricide molecules and/or combinations to prevent pet infestation by ectoparasites. Trends Parasitol.

[10] Wengenmayer C, Williams H, Zschiesche E, Moritz A, Langenstein J, Roepke RKA (2014). The speed of kill of fluralaner (Bravecto™) against *Ixodes ricinus* ticks on dogs. Parasit Vectors.

[11] Dantas-Torres F, Latrofa MS, Annoscia G, Giannelli A, Parisis A, Otranto D (2013). Morphological and genetic diversity of *Rhipicephalus sanguineus sensu lato* from the New and Old Worlds. Parasit Vectors.

[12] Dantas-Torres F (2008). The brown dog tick, *Rhipicephalus sanguineus* (Latreille, 1806) (Acari: Ixodidae): From taxonomy to control. Vet Parasitol.

[13] Dantas-Torres F (2010). Biology and ecology of the brown dog tick. Rhipicephalus sanguineus Parasit Vectors.

[14] Demma LJ, Traeger MS, Nicholson WL, Paddock CD, Blau DM, Eremeeva ME (2005). Rocky Mountain spotted fever from an unexpected tick vector in Arizona. N Engl J Med.

[15] Otranto D, Huchet J-B, Giannelli A, Callou C, Dantas-Torres F (2014). The enigma of the dog mummy from Ancient Egypt and the origin of ‘*Rhipicephalus sanguineus*’. Parasit Vectors.

[16] Dryden MW (2009). Flea and tick control in the 21st century: challenges and opportunities. Vet Dermatol.

[17] UK DEFRA. Summary of product characteristics, Advantix Spot-on solution for dogs over 25 kg. 2015. Available from: URL: www.vmd.defra.gov.uk/productinformationdatabase/spc_documents/spc_151145.doc. Accessed 1 Aug 2016.

[18] Taenzler J, Liebenberg J, Roepke RKA, Heckeroth AR (2015). Prevention of transmission of *Babesia canis* by *Dermacentor reticulatus* ticks to dogs treated orally with fluralaner chewable tablets (Bravecto™). Parasit Vectors.

[19] Beugnet F, Halos L, Larsen D, Labuschagné M, Erasmus H, Fourie J (2014). The ability of an oral formulation of afoxolaner to block the transmission of *Babesia canis* by *Dermacentor reticulatus* ticks to dogs. Parasit Vectors.

[20] Honsberger NA, Six RH, Heinz TJ, Weber A, Mahabir SP, Berg TC (2016). Efficacy of sarolaner in the prevention of *Borrelia burgdorferi* and *Anaplasma phagocytophilum* transmission from infected *Ixodes scapularis* to dogs. Vet Parasitol.

[21] Fourie JJ, Luus HG, Stanneck D, Jongejan F (2013). The efficacy of Advantix to prevent transmission of *Ehrlichia canis* to dogs by *Rhipicephalus sanguineus* ticks. Parasite.

